# A phase Ib/II randomized, open-label drug repurposing trial of glutamate signaling inhibitors in combination with chemoradiotherapy in patients with newly diagnosed glioblastoma: the GLUGLIO trial protocol

**DOI:** 10.1186/s12885-023-11797-z

**Published:** 2024-01-15

**Authors:** Maximilian Mastall, Patrick Roth, Andrea Bink, Angela Fischer Maranta, Heinz Läubli, Andreas Felix Hottinger, Thomas Hundsberger, Denis Migliorini, Adrian Ochsenbein, Katharina Seystahl, Lukas Imbach, Tibor Hortobagyi, Leonhard Held, Michael Weller, Hans-Georg Wirsching

**Affiliations:** 1 Department of Neurology, Clinical Neuroscience Center and Brain Tumor Center, University Hospital Zurich, Frauenklinikstrasse 26, Zurich, CH-8091 Switzerland; 2 Department of Neurology, University of Zurich, Zurich, Switzerland; 3 Department of Neuroradiology, Clinical Neuroscience Center, University Hospital Zurich, Zurich, Switzerland; 4 Department of Hematology and Oncology, Cantonal Hospital Graubünden, Chur, Switzerland; 5 Division of Medical Oncology, University Hospital Basel, Basel, Switzerland; 6 Department of Oncology, Centre Hospitalier Universitaire Vaudois, Lausanne, Switzerland; 7 Department of Neurology and Medical Oncology, Cantonal Hospital St. Gallen, St. Gallen, Switzerland; 8 Department of Oncology, Hopitaux Universitaires de Genève, Geneva, Switzerland; 9 Department of Medical Oncology, Inselspital, Bern University Hospital, Bern, Switzerland; 10 Department of Neurology and Neurorehabilitation, Cantonal Hospital Lucerne, Lucerne, Switzerland; 11 Swiss Epilepsy Center – Klinik Lengg, Zurich, Switzerland; 12 Department of Neuropathology, University Hospital Zurich, Zurich, Switzerland; 13 Department of Biostatistics, Epidemiology Biostatistics and Prevention Institute, University of Zurich, Zurich, Switzerland

**Keywords:** Epilepsy, Gabapentin, Sulfasalazine, Memantine, Cancer neuroscience

## Abstract

**Background:**

Glioblastoma is the most common and most aggressive malignant primary brain tumor in adults. Glioblastoma cells synthesize and secrete large quantities of the excitatory neurotransmitter glutamate, driving epilepsy, neuronal death, tumor growth and invasion. Moreover, neuronal networks interconnect with glioblastoma cell networks through glutamatergic neuroglial synapses, activation of which induces oncogenic calcium oscillations that are propagated *via* gap junctions between tumor cells. The primary objective of this study is to explore the efficacy of brain-penetrating anti-glutamatergic drugs to standard chemoradiotherapy in patients with glioblastoma.

**Methods/design:**

GLUGLIO is a 1:1 randomized phase Ib/II, parallel-group, open-label, multicenter trial of gabapentin, sulfasalazine, memantine and chemoradiotherapy (Arm A) versus chemoradiotherapy alone (Arm B) in patients with newly diagnosed glioblastoma. Planned accrual is 120 patients. The primary endpoint is progression-free survival at 6 months. Secondary endpoints include overall and seizure-free survival, quality of life of patients and caregivers, symptom burden and cognitive functioning. Glutamate levels will be assessed longitudinally by magnetic resonance spectroscopy. Other outcomes of interest include imaging response rate, neuronal hyperexcitability determined by longitudinal electroencephalography, Karnofsky performance status as a global measure of overall performance, anticonvulsant drug use and steroid use. Tumor tissue and blood will be collected for translational research. Subgroup survival analyses by baseline parameters include segregation by age, extent of resection, Karnofsky performance status, O^6^-methylguanine DNA methyltransferase *(MGMT)* promotor methylation status, steroid intake, presence or absence of seizures, tumor volume and glutamate levels determined by MR spectroscopy. The trial is currently recruiting in seven centers in Switzerland.

**Trial registration:**

NCT05664464. Registered 23 December 2022.

## Background

Glioblastoma is one of the deadliest cancer entities, with a median overall survival in the range of just one year in population-based studies [1, 2]. The standard of care is confined to maximum safe tumor resection followed by chemoradiotherapy with the alkylating agent temozolomide and maintenance temozolomide therapy [3–5], with or without electromagnetic fields applied via scalp electrodes [6]. Tumor recurrence invariably occurs and therapeutic options are then limited [5]. Therefore, there is an urgent medical need for improved therapeutic options for patients with glioblastoma especially in the first line treatment.

The discovery of electrochemically active, oncogenic neuroglial networks in glioblastoma has sparked attempts to pharmacologically disrupt these networks [7, 8]. Glioblastoma cells interconnect to form electrochemically active networks via gap junctions [9] and these glioma cell networks synaptically integrate into neuronal circuits [10, 11]. Oncogenic calcium oscillations of tumor cell networks are activated by autonomously oscillating hub cells [12] which are present mainly in the tumor core and through activation of glutamatergic neuroglial synapses within the glioblastoma infiltration zone [10, 11]. Remodeling of distant neuronal networks can activate tumor cell networks in a vicious cycle, including through epileptic activity and by activity-dependent shedding of neuronal growth factors [13, 14]. Of note, a recent study of long-term electroencephalographic recordings in glioblastoma patients suggests high rates of sub-clinical epileptic activity which may contribute to inferior survival [15, 16].

Glioblastoma cells also synthesize large amounts of the excitatory neurotransmitter glutamate from α-ketoglutarate via branched chain amino acid transaminase-1 (BCAT-1) [17] which is released into the tumor microenvironment at high concentrations via the glutamate-cystine antiporter system x_c_ [18, 19]. This non-synaptic glutamate release may drive glioma cell invasion [20] and will likely enhance the hyperexcitability and thus the oncogenic activity of neuroglial networks [21].

Several brain-penetrating, anti-glutamatergic drugs that are clinically approved for other indications have been identified, including (i) the anti-epileptic drug gabapentin, which interferes with the binding of branched-chain amino acids to BCAT-1 and inhibits thrombospondin-1 signaling by blocking the thrombospondin receptor α2δ-1 [17, 22, 23], (ii) the anti-inflammatory drug sulfasalazine, which inhibits glutamate secretion by blocking the cystine-glutamate exchanger system x_c_ [24], and (iii) the cognitive enhancer memantine, which blocks N-methyl-D-aspartate (NMDA) type glutamate receptors, thereby inhibiting tumor cell invasion and neuroglial synapse formation [25, 26].

The omnipresence and pleiotropic functions of glutamate in glioblastoma lend rationale for a combined anti-glutamatergic therapeutic approach. The well-documented tolerability of some of these drugs supports the feasibility of a drug repurposing approach in combination with standard chemoradiotherapy. There is limited commercial interest in exploring the activity of these drugs as anti-cancer agents.

## Methods

### Study objectives

The primary objective of this study is to evaluate whether the addition of gabapentin, sulfasalazine and memantine to standard chemoradiotherapy compared to chemoradiotherapy alone improves outcome of patients with newly diagnosed glioblastoma as determined by progression-free survival at 6 months. Secondary objectives are to determine tolerability, response rates as defined by the Response Assessment in Neuro-Oncology (RANO) working group [27], progression-free survival, overall survival, seizure-free survival, patient quality of life assessed by the European Organization for Research and Treatment of Cancer Quality of Life Questionnaire C30 and Brain Tumor Module 20 (EORTC-QLQ-C30/BN20) [28, 29], caregiver quality of life utilizing the CareGiver Oncology Quality of Life Questionnaire (CarGOQoL) [30], symptom burden measured by the MD Anderson Symptom Inventory Brain Tumor (MDASI-BT) [31] and by the Neurological Assessment in Neuro-Oncology (NANO) scale [32], cognitive functioning assessed by the Montreal Cognitive Assessment (MoCA) test [33], tumor glutamate levels estimated by magnetic resonance spectroscopy as well as anticonvulsant drug and steroid use.

### Trial design

**Fig. 1:**
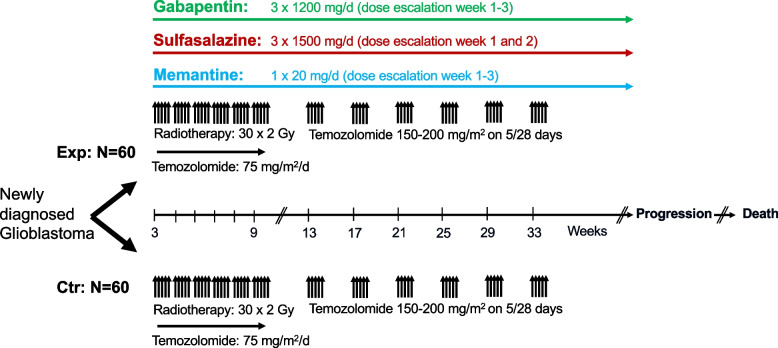
Schematic of the study design. Exp, experimental arm; Ctr, standard of care control arm

This study is an open-label, randomized, multicenter, phase Ib/II clinical trial. Following informed consent, patients who meet eligibility criteria will be randomly allocated in a 1:1 fashion to receive either a triple glutamate-targeted treatment with gabapentin, sulfasalazine and memantine plus chemoradiotherapy with temozolomide or chemoradiotherapy alone (Fig. 1). A total of 120 patients will be randomized with 60 participants in each study arm. The allocation sequence will be generated in advance using stratified block randomization with varying block sizes. Randomization will be stratified by extent of resection (gross total versus subtotal resection or biopsy). Post hoc central neuropathology review will be conducted for quality assurance. Randomized patients will enter the treatment phase and will be followed-up until death. Tumor progression will be assessed by contrast-enhanced magnetic resonance imaging every 3 months. An epileptic seizure assessment questionnaire will be filled in at every study visit and routine electroencephalography will be performed every 3 months to assess epileptic seizure control and neuronal hyperexcitability. Data bank closure will be 6 months after the last participant was randomized.

### Patient cohort

Patients are recruited at 7 sites in Switzerland (University Hospital Zurich; University Hospital Geneva; University Hospital Basel; Cantonal Hospital Lucerne; University Hospital Bern; Cantonal Hospital St. Gallen; Cantonal Hospital Graubünden). The first patient was enrolled in January 2023.

### Inclusion criteria

Newly diagnosed supratentorial glioblastoma according to the 2021 WHO Classification of central nervous system tumors [34]; eligible for standard chemoradiotherapy with temozolomide (hypofractionated radiotherapy regimen not allowed); age ≥ 18 years; Karnofsky performance status of ≥ 70; normal kidney and liver function; normal hematologic parameters.

### Exclusion criteria

Intent to be treated with tumor-directed therapy other than chemoradiotherapy; pregnant or breast feeding women; intention to become pregnant or father a child during study course; lack of safe contraception; clinically significant concomitant disease; known or suspected non-compliance, drug or alcohol abuse; inability to follow the procedures of the study; participation in another study with an investigational drug; contraindication for gadolinium-enhanced MRI; any prior radiotherapy of the brain; active malignancy that may interfere with the study treatment; abnormal ECG with QTc > 450 ms; previous intolerance reactions to one of the study drugs; intolerance reactions to sulfonamides or salicylates; acute intermittent porphyria; known glucose-6-phosphate dehydrogenase deficiency; concomitant therapy with digoxin, ciclosporin, methotrexate; history of exfoliative dermatitis, Stevens Johnson syndrome, toxic epidermal necrolysis, drug rash with eosinophilia and systemic symptoms (DRESS) syndrome or renal tubular acidosis.

### Study treatment

Study treatment includes oral gabapentin, sulfasalazine and memantine in the experimental study arm. Temozolomide and radiotherapy are standard of care and given to patients in both arms. Dosing of the investigational drugs in Arm A will be sought up to the maximum approved dose and will be reevaluated in an interim safety analysis after 20 patients have been randomized into the experimental arm. The investigational drugs will be given until tumor progression or withdrawal, whichever occurs first. Dosing will be reduced for at least one week in case of CTCAE grade 3 and permanently discontinued in case of CTCAE grade 4 drug-related toxicity, respectively. If toxicity is resolved to CTCAE grade 0–1, reescalation to higher dose levels is allowed. Permanent discontinuation of one out of the three investigational drugs for toxicity will not be considered treatment failure. Permanent discontinuation of two or more drugs will be considered treatment failure. For discontinuation, investigational drugs will be tapered following the reverse schedule as for the initial dosing.

### Gabapentin

Gabapentin is approved for the treatment of epilepsy and neuropathic pain. The definite mechanism of action by which gabapentin exerts anti-convulsant and analgetic effects has not been fully clarified. Oral gabapentin will be given at a dose of 3 × 300 mg/day in week 1, 3 × 600 mg/day in week 2, 3 × 900 mg/day in week 3 and 3 × 1200 mg/day from week 4 onwards. The most common adverse events related to gabapentin include neurological symptoms, e.g. ataxia, somnolence, dizziness, vertigo, tremor, diplopia, amblyopia and nystagmus. Dosing will be permanently discontinued if DRESS syndrome attributed to gabapentin occurs.

### Sulfasalazine

Sulfasalazine is approved for the treatment of ulcerative colitis and rheumatoid arthritis. Oral sulfasalazine will be given at a dose of 3 × 500 mg/day in week 1, 3 × 1000 mg/day in week 2 and 3 × 1500 mg/day from week 3 on. Dosing will be reduced if hematologic, liver or renal toxicity occurs and will be permanently discontinued if Lyell syndrome, Stevens Johnson syndrome or DRESS syndrome occurs.

### Memantine

Memantine is approved for the treatment of Alzheimer’s disease. Oral memantine will be given at a dose of 1 × 5 mg/day in week 1, 1 × 10 mg/day in week 2, 1 × 15 mg/day in week 3 and 1 × 20 mg/day from week 4 onwards). Higher grade toxicity from memantine is overall rare.

### Radiotherapy

Patients will receive radiotherapy in daily fractions of 1.8 - 2 Gy given 5 days per week over 6-7 weeks, for a total dose of 60 Gy delivered in 30 - 33 fractions. Radiotherapy will be administered concomitantly with temozolomide and in the experimental arm also with the investigated drugs. Target volume delineation will be based on postoperative MRI scans (minimum: T1 native and T1 + Gadolinium, T2/FLAIR; axial orientation) obtained for treatment planning taking pre-operative MRI into consideration as well. Every effort is made to deliver the full dose to all patients. Up to 7 days of treatment interruption are permitted for any reason.

### Temozolomide

Temozolomide will be administered during radiotherapy at a dose of 75 mg/m^2^ daily at 7 days per week. This is followed by maintenance therapy with up to 6 cycles temozolomide at 150 to 200 mg/m^2^ for 5 consecutive days every 4 weeks, beginning 4 weeks after the end of radiotherapy [3]. The most common expected toxicity is myelosuppression. If adverse events persist, treatment will be delayed by 1 week for up to 4 consecutive weeks, after which temozolomide will be discontinued, if adverse events have not resolved to ≤ grade 1.

### Statistical considerations

We considered an increase of the progression-free survival rate at 6 months (PFS-6) rate by 20% a clinically meaningful result that would warrant further exploration in a phase III clinical trial (assuming 50% survival rate following chemoradiotherapy alone and 70% with chemoradiotherapy plus gabapentin, sulfasalazine and memantine). At a power of 80% and a one-sided significance level of 10%, allowing a 10% drop-out rate, 120 patients need to be recruited (60 patients per arm) to detect this difference. The primary outcome will be assessed using a one-sided comparison of the PFS-6 proportion of patients in the two treatment arms at significance level 10% and a 90% confidence interval for the risk difference. Subgroup analyses will be based on two-sided interaction tests at a significance level 5%. The 8 subgroup analyses will not be adjusted for multiplicity and potential findings will be interpreted exploratively.

## Discussion

The recent discovery of glutamatergic neuroglial synapses between peritumoral neurons and glioma cells has sparked cancer neuroscience as a rapidly evolving research field [8, 35]. Several pre-clinical studies suggest that pharmacologic interference with these synapses may inhibit glioma growth and invasion [10, 11, 22, 35]. Hyperexcitability of neuronal networks and tumor-associated epilepsy are deemed drivers of neuroglial signaling [22, 36]. Finally, non-synaptic secretion of glutamate into the tumor microenvironment by glioblastoma cells may likewise contribute to hyperexcitability and glioblastoma progression [20, 37]. The randomized GLUGLIO trial explores the efficacy of a triple anti-glutamatergic combination of gabapentin, sulfasalazine and memantine to address whether glutamate may be exploited as a therapeutic lever.

Gabapentin reduces glutamate synthesis through inhibition of BCAT-1 [17] and, through the inhibition of thrombospondin-1 receptor α2δ-1, has been found recently to reduce functional connectivity of glioma and neuronal networks by inhibiting synaptogenesis and thus reducing tumor cell proliferation [22]. Moreover, the anti-convulsant effect of gabapentin alone may be beneficial to patients since a contribution of epilepsy to glioblastoma progression has been suggested by several pre-clinical and clinical studies [16, 22, 36], and long-term electroencephalography suggests that sub-clinical epileptic activity is common [15].

Along the same lines, a reduction in tumor-associated epilepsy has also been demonstrated for the inhibitor of the glutamate-cystine antiporter system x_c_ by sulfasalazine [37, 38]. A decrease of peritumoral glutamate after a single sulfasalazine administration has been documented in glioblastoma patients utilizing magnetic resonance spectroscopy [18].

Memantine may inhibit NMDA receptor-dependent synapse formation between neurons and tumor cells, interfering with similar processes as in long-term potentiation during physiologic memory formation [39] and as has been demonstrated in synapse formation between neurons and brain metastatic cancer cells [26]. Moreover, neuroprotective effects of NMDA receptor inhibition may enhance neurocognitive function, similar to the indication of memantine in the treatment of Alzheimer’s dementia [40].

Of the investigational medical products tested in the GLUGLIO trial, only two small, uncontrolled clinical studies have thus far sought to explore the efficacy of sulfasalazine and memantine, respectively: One study of monotherapy with sulfasalazine in glioblastoma patients with advanced disease has been terminated for lack of efficiency following the inclusion of 8 patients [41]. In an early phase clinical trial, memantine in combination with temozolomide with or without mefloquin and metformin was administered to patients with newly diagnosed glioblastoma and memantine was overall well tolerated [42]. However, the exploratory efficacy results of this trial are difficult to interpret, because there was no standard of care control arm, sample size per treatment arm was small and survival was not reported by treatment arm or excluding patients with isocitrate dehydrogenase-mutant astrocytomas.

Two ongoing phase I/II clinical trials seek to explore pharmacological interference with neural circuits and tumor cell networks in glioblastoma. The first trial conducted by the Neuro-Oncology Working Group of the German Cancer Society investigates meclofenamate as a means to disrupt gap junctions within tumor-microtube networks in recurrent glioblastoma, the primary endpoint being safety and efficacy measured by incidence of dose-limiting toxicities and progression-free survival, respectively (EudraCT 2021-000708-39). The second trial evaluates biological effects of perampanel, a non-competitive antagonist of AMPA-receptors, on neuron-tumor interactions in a pre-surgery setting (EudraCT 2023-503938-52). Another report of ten glioma patients treated with perampanel for intractable epilepsy found at best minor effects on tumor growth based on MR images [43]. However, the small cohort size and inclusion of various glioma entities limits the interpretability of this study with respect to anti-tumor efficacy. Other than the GLUGLIO trial, no randomized clinical trials or uncontrolled studies with efficacy endpoints addressing the interplay of neuronal networks and glioblastoma cells have been registered by 11/2023.

Whether or not epilepsy is causally related to survival of glioblastoma patients is not known. In fact, epilepsy has been proposed as an indicator of longer survival of glioblastoma patients [44], albeit retrospective analysis of survival associations with epilepsy are difficult to assess for several reasons, e.g. glioblastomas becoming symptomatic due to epilepsy as compared to such becoming symptomatic from other neurological deficits may be diagnosed earlier during the disease course, and rates of complete resection may be higher due to cortical tumor location and smaller tumor size [45]. One retrospective study therefore employed time-dependent multivariate analyses to analyze associations of epilepsy with survival of glioblastoma patients and supported the notion of unfavorable effects of epilepsy [16]. Finally, anticonvulsant therapy with valproic acid or levetiracetam was not associated with overall survival of glioblastoma patients in a post hoc analysis of a large merged cohort derived from different phase 3 clinical trials [46], but such analyses have limitations since only drug use at distinct timepoints could be analyzed and extent of drug exposure therefore remains uncertain.

The GLUGLIO trial addresses some of these issues, since pre-specified subgroup analyses, albeit with low power, try to address survival separately among patients with or without epilepsy will enhance our understanding of whether or not anti-convulsant therapy may indeed be beneficial for glioblastoma patients who do not suffer from clinically apparent epilepsy. The secondary objective of the GLUGLIO trial to explore epileptic activity and the conduct of serial EEG recordings will also help to better understand the postulated interplay between epilepsy and tumor progression.

A major limitation of the GLUGLIO trial is the small sample size, requiring one-sided hypothesis testing and setting the significance level to 10%. Small sample size will moreover limit the sensitivity of pre-defined subgroup survival analyses and of putative signal seeking post hoc analyses. Moreover, if efficacy of the addition of triple anti-glutamatergic therapy will indeed be observed in this trial, the combination approach precludes the definite assignment of efficacy to either of the individual drugs, thus compromising the design of a putative phase III follow-up trial. Additional pre-clinical studies in relevant tumor models will therefore be required. However, the rationale for the combination approach over testing a single drug was that combined targeting of glutamate synthesis (gabapentin), secretion (sulfasalazine) and signaling (memantine) may have additive effects, and that the lack of an efficacy signal would in reverse lend strong rationale against glutamate-targeted treatment approaches in the future.

The GLUGLIO trial is currently ongoing and first results are expected by the end of 2026.

## Data Availability

No datasets were generated or analysed during the current study.

## Abbreviations

BCAT-1: Branched chain amino acid transaminase-1
DRESS: Drug rash with eosinophilia and systemic symptoms
ESAQ: Epileptic seizure assessment questionnaire
Gy: Gray
NMDA: N-methyl-D-aspartate
PFS: Progression-free survival
RANO: Response Assessment in Neuro-Oncology
